# Rare “ileum–ileum–colon type” adult intussusception caused by Meckel’s diverticulum inversion: A case report

**DOI:** 10.1097/MD.0000000000041237

**Published:** 2025-01-03

**Authors:** Yi-Hu Mao, Li-Bin Huang, Qi Jia, Shu-Jun Li, Qian Zeng, Jian Yang, Cai-Jun Yang, Qi Pu, Xue-Ping Liu

**Affiliations:** a Department of Gastrointestinal Surgery, The People’s Hospital of Lezhi, Lezhi, China; b Division of Gastrointestinal Surgery, Department of General Surgery, West China Hospital of Sichuan University, Sichuan Province, China; c Department of Pathology, The People’s Hospital of Lezhi, Lezhi, China.

**Keywords:** adult, ileum–ileum–colon, intussusception, Meckel’s diverticulum

## Abstract

**Rationale:**

Ileum–ileum–colon intussusception constitutes a small proportion of the classification of intussusception. Both adult intussusception and Meckel’s diverticulum are rare clinical entities, with few reports documenting Meckel’s diverticulum inversion leading to ileum–ileum–colon intussusception in adults.

**Patient concerns:**

A 33-year-old Chinese male presented with intermittent abdominal pain persisting for 1 month, that had intensified over the preceding day.

**Diagnoses:**

Abdominal computed tomography revealed intussusception, suspected to be secondary to a small intestinal lipoma.

**Interventions:**

Emergency laparotomy was performed, during which the ileum, located approximately 70 cm from the ileocecal region, was found to be invaginated into the terminal ileum and subsequently into the ascending colon, reaching the hepatic flexure. An inverted Meckel’s diverticulum was at the forefront of the intussusception. Surgical resection of the diverticulum, terminal ileum, and cecum was performed, followed by an end-to-side anastomosis of the ileum and colon.

**Outcomes:**

The patient was discharged on postoperative day 7 without complications. During a 1-month follow-up, the patient reported no discomfort and exhibited normal bowel movements.

**Lessons:**

Adult intussusception of the “ileum–ileum–colon type” resulting from inverted Meckel’s diverticulum is exceedingly rare and poses challenges for preoperative diagnosis. Prompt surgical intervention can lead to favorable outcomes in patients. During surgery, the initial step should involve attempting reduction of the intussusception while ensuring that the intestine is preserved as much as possible to maintain intestinal function.

## 1. Introduction

Intussusception involves invagination of 1 segment of the intestine and its mesentery into another segment. While common in children, it is extremely rare in adults, with adult intussusception accounting for approximately 5% of all cases.^[1]^ Intestinal intussusception can be categorized into ileum–cecum, ileum–colon, ileum–ileum–colon, ileum–ileum, and colon–colon, based on the insertion site. The ileum–colon type is the most frequent, whereas the ileum–ileum–colon-type is very rare.^[2]^

Meckel’s diverticulum (MD) is a congenital anomaly resulting from incomplete retraction of the vitelline duct during embryonic development. Typically located on the antimesenteric border of the ileum within 100 cm of the ileocecal valve, MD has an incidence rate of approximately 0.3% to 2.9%. Similar to intussusception, MD is more prevalent in children and rarer in adults.^[3]^ Inverted Meckel’s diverticulum is a rare clinical presentation, with adult intussusception caused by MD inversion being extremely uncommon.^[4,5]^

Previous reports on MD inversion leading to adult intussusception primarily described the ileum–ileum type, with scarcely any reports of the ileum–ileum–colon type. This type is characterized by insertion of the ileum into the terminal ileum and subsequently into the colon. We present a rare case of adult ileum–ileum–colon intussusception caused by MD inversion that was initially misdiagnosed as lipoma on preoperative computed tomography (CT).

## 2. Case presentation

### 2.1. Patient information

A 33-year-old Chinese male presented to the gastrointestinal surgery department with a 1-month history of recurrent upper abdominal distension and pain exacerbated by eating, accompanied by heartburn, belching, nausea, and vomiting. There was no fever, diarrhea, or limb edema. A prior medication regimen provided only slight relief. The patient had no history of disease or surgery.

### 2.2. Physical examination

The patient exhibited poor mental state, no jaundice, and no cardiopulmonary abnormalities. His vital signs were as follows: temperature, 36.6°C, pulse 84/minute, respiration 21/minute, and blood pressure, 132/76 mm Hg. The patient had a flat abdomen with pressure pain around the navel and epigastrium without rebound pain or muscle tension.

### 2.3. Diagnostic testing

**Figure 1.**
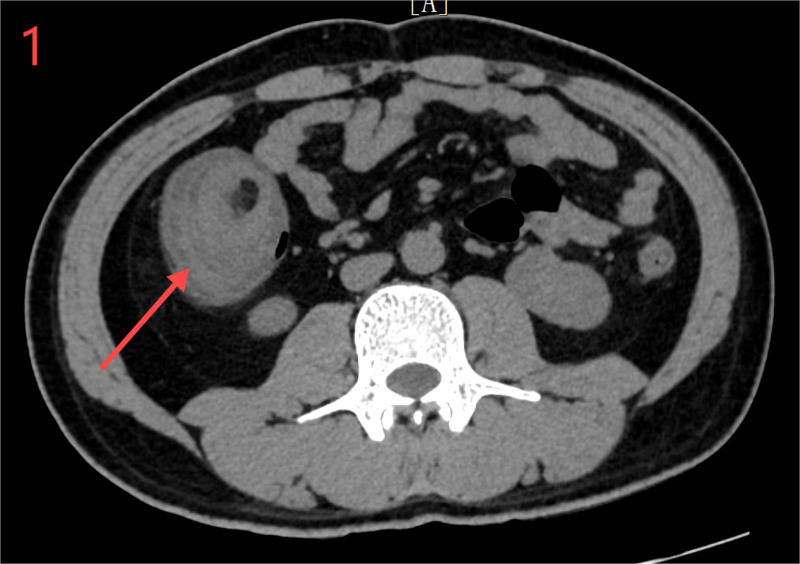
Computed tomography image (transverse plane) shows concentric circle sign in ascending colon (red arrow).

**Figure 2.**
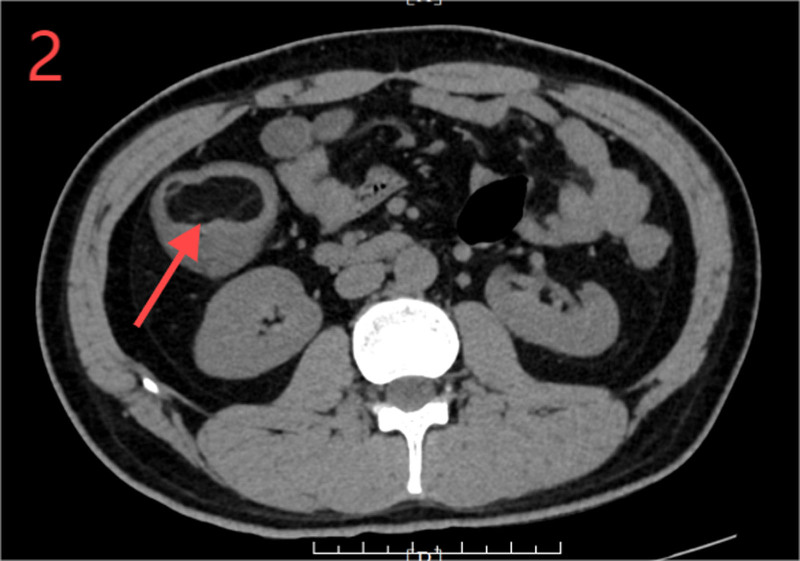
Computed tomography image (transverse plane) shows a circular fat density mass in the lumen of the ascending colon.

Laboratory tests showed a white blood cell count of 7.71 × 10^9^/L and neutrophil count of 8.9 × 10^9^/L, with 86.40% neutrophils. CRP, liver and kidney functions, electrolytes, coagulation profiles, and fecal occult blood were normal, and emergency abdominal CT revealed concentric changes in the ascending colon, with a portion of the small intestine, mesenteric blood vessels, and adipose tissue located within it (Fig. 1). Gas shadows were observed beneath the mucosa of the ascending colon, and the walls of the ascending and right transverse colon as well as some small intestines were swollen with a cloudy surrounding fat layer. Fluid accumulation and multiple small lymph nodes were noted near the mesenteric root and the appendix was not clearly visible, suggesting intussusception with surrounding inflammation. A nodule with circular fat density (~4.3 cm) and a slightly higher edge density was observed in the ascending colon, suggesting a lipoma (Fig. 2).

### 2.4. Therapeutic intervention, follow-up, and outcomes

**Figure 3.**
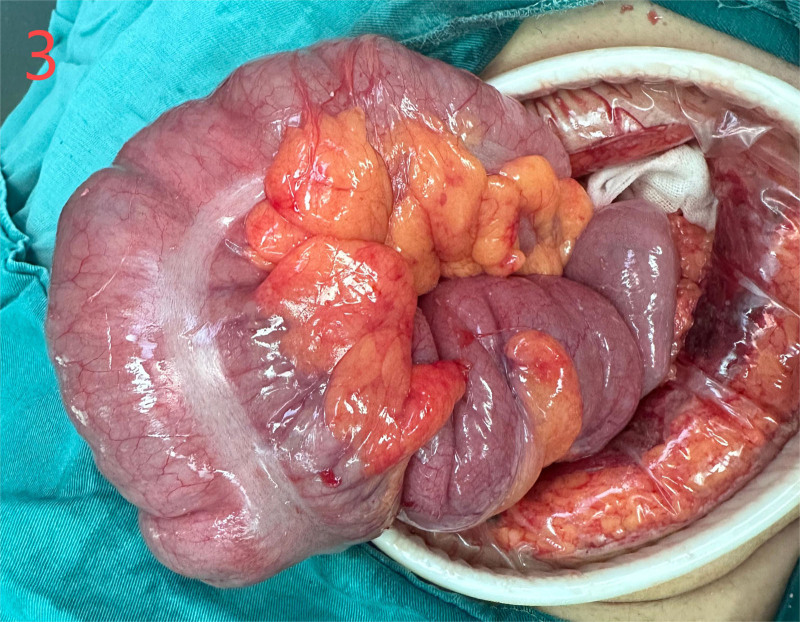
Intraoperative findings: The insertion of the ileum into the terminal ileum and then into the colon, severe edema in the terminal ileum.

**Figure 4.**
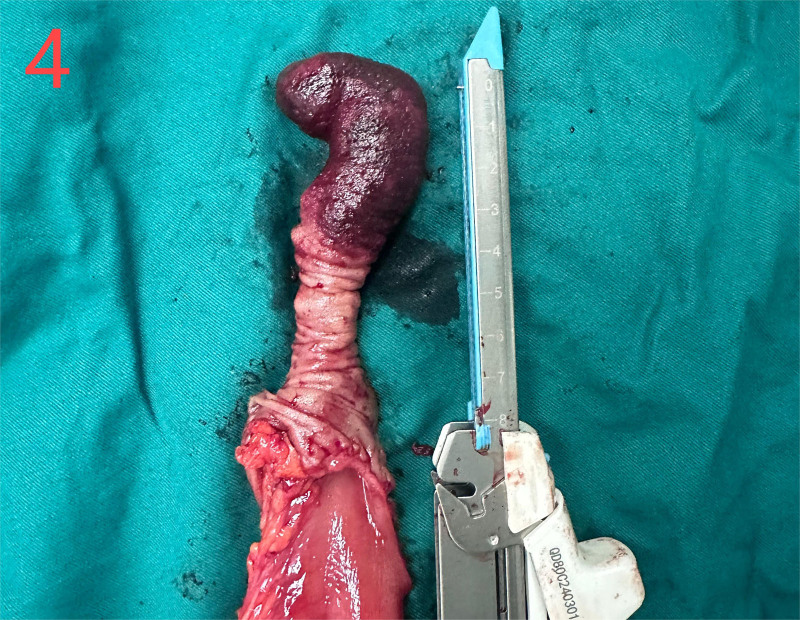
The inverted Meckel’s diverticulum of the segmental resection of the intestine, (2.5 cm × 8 cm).

**Figure 5.**
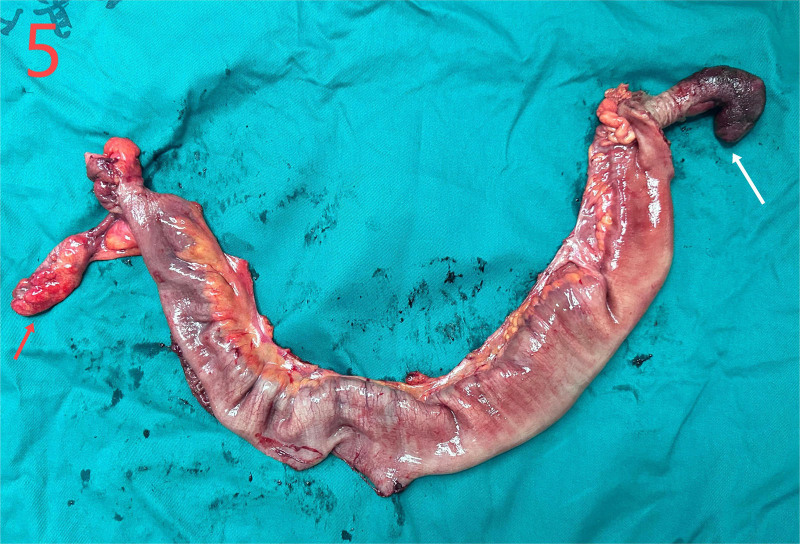
Gross specimen of the intestinal resection, appendix (red arrow), the inverted Meckel’s diverticulum (yellow arrow).

**Figure 6.**
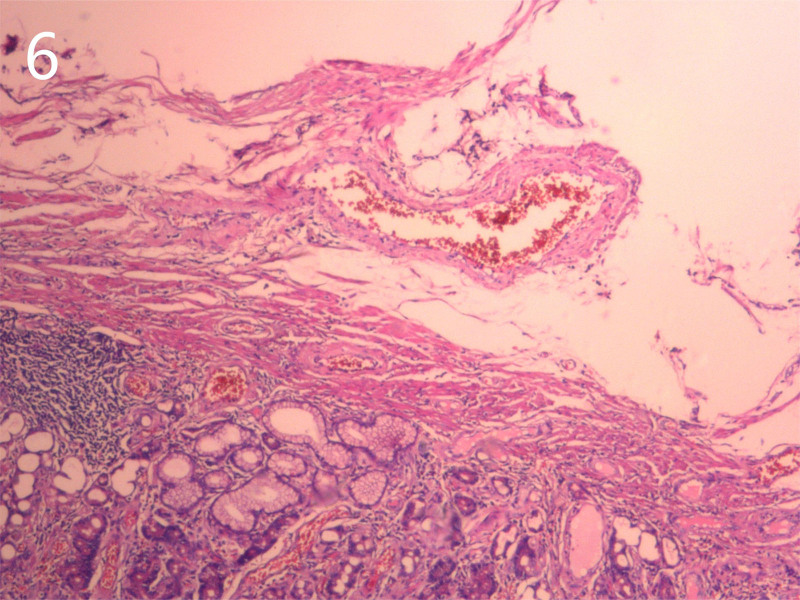
Histopathology reveals weak muscle layer in the small intestine wall and lymphocytes in the interstitium.

He was diagnosed with the following conditions: Intussusception; intestinal obstruction; emergency laparotomy under general anesthesia revealed that the ileum was inserted into the distal ileum and folded into the ascending colon through the ileocecal valve (Fig. 3). The intussusception extended to the hepatic flexure with mild edema of the intestinal wall. The terminal ileum, which was approximately 20 cm from the ileocecal valve, was highly congested and swollen. Due to intussusception and the resulting intestinal rupture, initial attempts to reduce intussusception during surgery failed. Opening of the ileocecal valve for decompression was unsuccessful. The terminal ileum was then opened longitudinally, allowing for successful reduction of the intussusception. The inserted segment was highly congested and edematous, with some ischemic intestinal walls and poor peristalsis, and the farthest end of the intussusception was approximately 70 cm from the ileocecal valve, with local serosa depressed into the intestinal lumen. A flexible mass (~2.5 × 8 cm) was palpable within the intestinal lumen. The proximal segment showed mild edema, but no necrosis. Surgical resection of the ileocecal region, including the appendix and terminal ileum (approximately 70 cm), was performed, followed by end-to-end anastomosis between the ileum and colon. The procedure had no complications. The gross specimens of the intestine and inverted Meckel’s diverticulum are shown in Figures 4 and 5. Histopathological examination revealed congestion and edema of the intestinal wall with dark red discoloration. Microscopic examination revealed mucosal bleeding, necrosis, lymphocyte infiltration, and interstitial edema. The protrusion exhibited thinning of the intrinsic muscle and lymphocyte infiltration, consistent with the diverticular changes (Fig. 6). The patient recovered uneventfully and was discharged 1 week postoperatively. A 1-month follow-up revealed no discomfort or abnormalities in bowel movements.

## 3. Discussion

Adult intussusception accounts for approximately 5% of all cases, which is significantly lower than that in children.^[1]^ Approximately 90% of adult intussusception cases are secondary to benign or malignant tumors as well as Meckel’s diverticulum.^[6]^ Intestinal intussusception is categorized by insertion site: ileum–cecum, ileum–colon, ileum–ileum–colon, ileum–ileum, and colon–colon. The ileum–colon type is the most common, whereas the ileum–ileum–colon type is rare.^[2]^

Adult Meckel’s diverticula are uncommon and may remain asymptomatic throughout an individual’s life, with approximately 4% exhibiting clinical symptoms.^[7]^ These symptoms are often nonspecific, and include abdominal pain, gastrointestinal bleeding, and intestinal obstruction. It is estimated that 4% to 14% of Meckel’s diverticula can lead to intussusception.^[8]^ The epidemiology of Meckel’s diverticulum resulting in intussusception remains unclear with only a limited number of reported cases. As of May 2021, fewer than 50 cases of Meckel’s diverticulum leading to intussusception have been documented in the database,^[5]^ and a very small proportion of these cases were diagnosed preoperatively.^[9]^ Consequently, ileum–ileum–colon-type adult intussusception caused by Meckel’s diverticulum inversion is particularly rare. The lack of clinically specific symptoms often complicates preoperative diagnosis, resulting in potential misdiagnosis.

Commonly used preoperative examination methods for intussusception include abdominal plain film, abdominal ultrasound, CT, and angiography. Currently, CT is a widely used clinical examination technique that can identify the site of intussusception, assess mesenteric blood supply, and detect the presence of intestinal necrosis prior to surgery, thereby enhancing the preoperative diagnosis rate. It is regarded as the gold standard for diagnosing intussusception.^[10]^ According to relevant literature, the accuracy of preoperative CT examination in diagnosing intussusception ranges from approximately 58% to 100%.^[2]^ However, preoperative diagnosis of Meckel’s diverticulum poses challenges. For patients suspected of having Meckel’s diverticulum before surgery, the examination methods may include ectopic mucosal imaging, digital subtraction angiography, CT plain scan, CT enhanced scan, CT small intestine contrast, capsule endoscopy, and double balloon enteroscopy. Among these, double-balloon enteroscopy demonstrates the highest accuracy, while the examination rate of plain CT scans is relatively low.^[11,12]^ Consequently, establishing a definitive diagnosis of adult intussusception caused by Meckel’s diverticulum inversion preoperatively is particularly challenging. Although CT examination can clarify the presence of intussusception, it cannot ascertain the underlying cause and may even lead to misdiagnosis. In this case, the patient was suspected to have a lipoma based on preoperative CT findings. This suspicion arose because adipose tissue may be present on the serosal surface of the Meckel’s diverticulum. When Meckel’s diverticulum inverts, this fat, along with some mesenteric fat, may accompany the diverticulum inversion, resulting in a CT image showing fat density at the center of the inverted diverticulum.^[13]^

Adult intussusception is predominantly secondary in nature and often associated with tumors, necessitating emergency surgical intervention. The specific surgical approach is determined by the disease location, its underlying cause, and the condition of the affected intestinal tract at the time of surgery. Currently, the majority of scholars advocate segmental resection and intestinal anastomosis in cases of intussusception. However, there is ongoing debate regarding the performance of intussusception reduction during surgery. If the cause of intussusception is a malignant tumor, intraoperative reduction may result in tumor dissemination,^[14]^ frequently requiring segmental or radical resection of the tumor.^[15]^ Conversely, if the cause is benign, such as lipoma or Meckel’s diverticulum, reduction may be attempted. In instances of ileum–ileum–colon intussusception, the affected segment is often longer, involving both the ileum and the colon. If reduction is unfeasible, right hemicolectomy is necessary. Excessive removal of intestinal segments can adversely affect the preservation of normal physiological function. Therefore, it is recommended that in cases of “ileum–ileum–colon” type adult intussusception secondary to Meckel’s diverticulum inversion, an initial attempt at reduction be made, followed by the excision of the diverticulum or diseased intestinal segment, with the goal of preserving as much intestinal length as possible to maintain normal physiological function.

The reason for the inversion of the Meckel’s diverticulum remains unclear. Pathologically, ectopic gastric or pancreatic tissue may be present within the diverticulum, with the ectopic gastric mucosa potentially secreting gastric acid that can lead to ulcer formation and subsequent gastrointestinal bleeding or perforation. Furthermore, there is often a lack of coordination between peristalsis of the ectopic mucosa and that of the surrounding non-ectopic mucosal tissue. This uncoordinated movement of the root, body, and base of Meckel’s diverticulum may contribute to its inversion.^[8]^ Additionally, the Meckel’s diverticulum is situated at the edge of the mesentery in the small intestine and lacks a role in anchoring the mesentery. As a result, it exhibits considerable mobility and can easily shift into adjacent intestinal cavities owing to intestinal peristalsis.^[9]^ The negative pressure generated by the passage of intestinal contents exerts a suction effect on Meckel’s diverticulum, further promoting its inversion towards the intestinal lumen.

## 4. Summary

In summary, the “ileum–ileum–colon type” of adult intussusception caused by Meckel’s diverticulum inversion is exceedingly rare. This condition presents with nonspecific clinical symptoms, complicating preoperative diagnosis and increasing the risk of misdiagnosis. Clinicians should consider the possibility of intussusception and Meckel’s diverticulum in adults presenting with intermittent abdominal pain. A preoperative CT scan can be instrumental in establishing a diagnosis, and timely surgical intervention can facilitate favorable recovery outcomes. During surgery, efforts should be focused on reducing intussusception while preserving as many normal intestinal segments as possible to maintain normal physiological functions in the intestine.

## Acknowledgments

The authors gratefully acknowledge all staff and nurses for their kind cooperation, and thank the patient for their kind cooperation.

## Author contributions

**Conceptualization:** Yi-Hu Mao

**Investigation:** Yi-Hu Mao.

**Methodology:** Yi-Hu Mao.

**Supervision:** Yi-Hu Mao.

**Visualization:** Yi-Hu Mao, Qi Jia, Shu-Jun Li, Qian Zeng, Jian Yang, Cai-Jun yang, Qi Pu, Xue-Ping Liu.

**Writing – original draft:** Yi-Hu Mao, Li-Bin Huang.

**Writing – review & editing:** Yi-Hu Mao, Li-Bin Huang.
